# Technological Resources to Prevent Cyberbullying During Adolescence: The Cyberprogram 2.0 Program and the Cooperative Cybereduca 2.0 Videogame

**DOI:** 10.3389/fpsyg.2018.00745

**Published:** 2018-05-16

**Authors:** Maite Garaigordobil, Vanesa Martínez-Valderrey

**Affiliations:** Faculty of Psychology, University of the Basque Country (UPV/EHU), Bilbao, Spain

**Keywords:** bullying, cyberbullying, intervention, adolescence, videogames

## Abstract

Bullying and cyberbullying have serious consequences for all those involved, especially the victims, and its prevalence is high throughout all the years of schooling, which emphasizes the importance of prevention. This article describes an intervention proposal, made up of a program (Cyberprogram 2.0 Garaigordobil and Martínez-Valderrey, 2014a) and a videogame (Cooperative Cybereduca 2.0 Garaigordobil and Martínez-Valderrey, 2016b) which aims to prevent and reduce cyberbullying during adolescence and which has been validated experimentally. The proposal has four objectives: (1) To know what bullying and cyberbullying are, to reflect on the people involved in these situations; (2) to become aware of the harm caused by such behaviors and the severe consequences for all involved; (3) to learn guidelines to prevent and deal with these situations: know what to do when one suffers this kind of violence or when observing that someone else is suffering it; and (4) to foster the development of social and emotional factors that inhibit violent behavior (e.g., communication, ethical-moral values, empathy, cooperation…). The proposal is structured around 25 activities to fulfill these goals and it ends with the videogame. The activities are carried out in the classroom, and the online video is the last activity, which represents the end of the intervention program. The videogame (www.cybereduca.com) is a trivial pursuit game with questions and answers related to bullying/cyberbullying. This cybernetic trivial pursuit is organized around a fantasy story, a comic that guides the game. The videogame contains 120 questions about 5 topics: cyberphenomena, computer technology and safety, cybersexuality, consequences of bullying/cyberbullying, and coping with bullying/cyberbullying. To evaluate the effectiveness of the intervention, a quasi-experimental design, with repeated pretest-posttest measures and control groups, was used. During the pretest and posttest stages, 8 assessment instruments were administered. The experimental group randomly received the intervention proposal, which consisted of one weekly 1-h session during the entire school year. The results obtained with the analyses of variance of the data collected before and after the intervention in the experimental and control groups showed that the proposal significantly promoted the following aspects in the experimental group: (1) a decrease in face-to-face bullying and cyberbullying behaviors, in different types of school violence, premeditated and impulsive aggressiveness, and in the use of aggressive conflict-resolution strategies; and (2) an increase of positive social behaviors, self-esteem, cooperative conflict-resolution strategies, and the capacity for empathy. The results provide empirical evidence for the proposal. The importance of implementing programs to prevent bullying in all its forms, from the beginning of schooling and throughout formal education, is discussed.

## Introduction: conceptualization, prevalence and prevention of bullying/cyberbullying

### Bullying and cyberbullying: conceptualization

Bullying is a specific form of school violence, where one or more attackers intentionally cause pain, harass, and repeatedly subject another classmate. When we talk about face-to-face or presential bullying, we refer to: (1) the existence of a defenseless victim, harassed by one or more assailants; (2) who carry out different types of aggressive face-to-face behavior toward the victim, aggressive physical behaviors aimed at the victim's body or property (hitting, pushing, breaking, hiding, or stealing the victim's objects…), aggressive verbal behaviors (giving nicknames, insulting, saying unpleasant things about the victim…), behaviors of social exclusion (not letting the victim participate, excluding, telling lies, or spreading false rumors about the victim so she will be rejected by others…), aggressive psychological behaviors (aimed at undermining the victim's self-esteem, creating insecurity and fear: threatening, blackmailing, laughing at him, humiliating him…; although it should be borne in mind that all types or forms of bullying have a psychological component); (3) it is maintained physical and mental violence; (4) the aggressors intend to harm, they are purposely cruel to make the victim suffer; (5) there is usually an inequality of power between the victim and the aggressors (physical, verbal, or psychological inequality); (6) these aggressive behaviors are repeated frequently, there is a dominion-submission relationship between the aggressor or aggressors and the victim that is maintained over time; and (7) in addition, the aggression not only produces pain when it occurs, as the victim feels sustained pain and anguish due to expectations of future attacks, aggressions, or humiliation that he or she anticipates suffering (Garaigordobil, 2017).

In recent years, other forms of bullying have emerged, such as cyberbullying, which consists of using information and communication technology (ICT)—mainly Internet and mobile phones—to practice peer harassment. Cyberbullying is bullying in digital format. What behaviors do cyberaggressors perform? A review of the behaviors identified by numerous authors (Aftab, 2010; Kowalski et al., 2010; Tokunaga, 2010; Garaigordobil, 2013, 2015) identifies the following: (1) sending insulting, threatening, disparaging, or intimidating messages through mobiles or e-mail (ugly, fat, everybody hates you, you should die, be careful; we're going to beat you up…); (2) making anonymous phone calls to frighten the victim; and/or making threatening, intimidating, insulting, or disparaging calls…; (3) manipulating photographs to ridicule or create a false image of the victim, which the aggressors distribute by mobile phone or internet; (4) excluding, isolating the victim from social networks (Facebook, WhatsApp, Instagram…); (5) stealing the victim's password, and impersonating her identity (for example, sending aggressive messages to the victim's contacts to anger them; violating the victim's privacy, changing her password to prevent her access to her email account…); (6) provoking the victim in chats, online games, virtual communities… to achieve her violent reaction, which they then denounce to the Service Manager so he will impede her access to that service; (7) creating a false profile of the victim and, for example, making explicit offers of sexual contacts, giving the victim's mobile phone as the contact…; (8) signing up on some websites with the victim's email address so he will continuously receive emails and SPAM…; (9) disseminating lies about the victim to harm her (false rumors, slander…); (10) disseminating secret or embarrassing information about the victim, for example, concerning his orientation to his sexual identity; (11) denigrating or badmouthing the victim on a website, a personal blog, a social network…; (12) making surveys to disparage the victim, for example, choosing her as the ugliest, the least intelligent, the fattest… and giving her the points or votes, which go to her email; (13) beating up or placing the victim in a humiliating situation, recording it on the mobile, and broadcasting the video via mobile or uploading it to YouTube (happy slapping)… (Garaigordobil, 2017).

Therefore, in a situation of bullying and cyberbullying, we can differentiate three roles: a victim, one or more aggressors, and various observers whose silence and passivity largely facilitate the perpetuation of the situation over time. Observers frequently do not speak out due to lack of empathy with the victim and, other times, out of fear that the aggressor will turn against them.

### Bullying and cyberbullying: prevalence

What percentage of students is suffering from bullying and cyberbullying? To answer this question, we carried out a review, using a variety of databases (PsycInfo, Psicodoc, Scopus, Dialnet, CSIC, Latindex, PsycArticles, Eric, Google Scholar…), which identified 309 epidemiological studies that have examined the prevalence of bullying and cyberbullying at the national and international level, since the first study carried out by Olweus (1973).

The review of the results of studies on bullying, both national (e.g., among others, Irakas-Sistema Ebaluatu Eta Ikertzeko Erakundea-Instituto Vasco de Evaluación e Investigación Educativa (ISEI-IVEI), 2009, 2012; Cerezo and Méndez, 2013; Garcia-Continente et al., 2013; Fernández-Montalvo et al., 2015; Navarro et al., 2015a,b; García-Fernández et al., 2016) and international (e.g., among others, Olweus, 2013; Ybarra et al., 2014; Bogolyubova et al., 2015; Hase et al., 2015; Malhi et al., 2015; McClanahan et al., 2015; Sumter et al., 2015; Pabian and Vandebosch, 2016; Safaria, 2016; Shin et al., 2016) shows an average percentage of frequent (severe) victimization that ranges approximately between 2 and 16%, but the percentage of students who suffer violent face-to-face behavior, albeit occasionally, exceeds 80% in some studies. In relation to the percentage of aggressors, studies show a range of severe aggressors between 2 and 12%, although in some studies, the percentage of occasional aggressors reaches 45%.

The review of studies of prevalence of cyberbullying, both national (e.g., among others, Irakas-Sistema Ebaluatu Eta Ikertzeko Erakundea-Instituto Vasco de Evaluación e Investigación Educativa (ISEI-IVEI), 2009, 2012; Buelga et al., 2010; Calvete et al., 2010; Estévez et al., 2010; Cerezo and Méndez, 2013; Gámez-Guadix et al., 2013; Garaigordobil, 2015; Navarro et al., 2015a,b) and international (e.g., among others, Olweus, 2013; Stewart et al., 2014; Ybarra et al., 2014; Hase et al., 2015; Sumter et al., 2015; Tsitsika et al., 2015; Wu et al., 2015; Pabian and Vandebosch, 2016; Safaria, 2016; Shin et al., 2016) reveals a mean percentage of serious or severe (very frequent) cybervictimization ranging approximately between 1 and 10%, but the percentage of students who suffer cyberbullying behavior, even if it is occasional, exceeds 60% in some studies.

As seen in the review, cyberbullying is an increasing phenomenon, as in every study carried out, higher percentages of cybervictimized students appear. This increase is occurring in part because children have access to new technologies (Internet, mobile phones…) at increasingly earlier ages; because their activities in cyberspace are increasingly more relevant as socialization and entertainment spaces; because, as they do not occur in a face-to-face situation, the aggressors have a lower perception of the harm to the victim; sometimes, they even consider their behavior like a game, as if they were interpreting a role of fiction; also, the perception of anonymity sometimes increases the feeling of impunity; and also due to the characteristics of the Internet, which facilitate the grouping of cyberaggressors and the production/dissemination of audiovisual materials. In relation to the percentage of cyberaggressors, some studies show a range of severe cyberaggressors between 1 and 8% although, in other studies, the percentage of occasional cyberaggressors reaches 70%.

The prevalence percentages of bullying and cyberbullying are not homogeneous but instead, they vary in the different studies. It is difficult to provide a specific figure that reflects their level of prevalence in children, adolescents, and young people. Regardless of whether there is more or less presence of the problem of abuse and its different forms in different countries, the research data are not easily comparable for several reasons. Different studies vary in terms of age (5–22 years in bullying, 7–25 years in cyberbullying), the assessment technique or tool employed, the type of behavior studied (especially in cyberbullying), or the time interval considered (some ask to what extent bullying was suffered in the past year, others in recent months, others do not establish any time limit). All this only allows us to offer a range of percentages of serious victimization (severe victims/cybervictims) and a much higher percentage of occasional victimization. However, the results of prevalence studies highlight that: (1) bullying/cyberbullying is a phenomenon that occurs in all countries and in all social classes; and (2) the problem is worthy of consideration, which allows us to emphasize the need for assessment, prevention, and intervention.

### Bullying and cyberbullying: prevention programs

Over the past decade, higher educational and social awareness toward the phenomenon of bullying has promoted an increase in preventive and palliative measures when it occurs. Although some of these prevention and intervention programs have been experimentally validated, a broad set of them still requires experimental validation processes that prove their consistency as suitable tools for the inhibition and decrease of bullying/cyberbullying.

In relation to the antibullying programs, we carried out a review. The results underscore the scarcity of interventions aimed at children of early ages. In addition, it is important to point out that the results of the antibullying programs are inconsistent. The effects vary greatly between programs, some have very weak effects, some do not even have any positive effects.

In this sense, some meta-analyses disagree with their effectiveness. For example, Ferguson et al. (2007) concluded that antibullying programs generate few effects in young participants, whereas the meta-analysis of Ttofi and Farrington (2011) showed that antibullying programs applied in school are effective. The meta-analysis of Ttofi and Farrington noted that, in general, these antibullying programs in school are effective to reduce bullying (on average by 20–23%) and victimization (by 17–20%). The improvement is increased as the participants in the intervention grow older. Programs that involve many hours of intervention for a long time interval are more effective, and also those that include meetings with parents (parent involvement), strong educational discipline (the use of disciplinary measures with the aggressors), cooperative learning, the use of anti-bullying videos, and supervision of recess. In the same direction, Menesini and Salmivalli (2017) considered that the programs in which the whole school is involved to prevent bullying are often successful. For these authors, student awareness about the important role of the group in the elimination of bullying is crucial.

The high prevalence of bullying and cyberbullying, with their serious consequences, emphasizes the need to develop resources aimed at preventing and reducing these behaviors of face-to-face, and cybernetic (digital bullying). Within this contextualization and justification, we propose this work, which consisted of the design and evaluation of the effects of an intervention made up of two tools (Cyberprogram 2.0 and Cooperative Cybereduca 2.0) which are intended to educate youngsters about the adequate use of the ICT, as well as to prevent and reduce bullying and cyberbullying.

## The intervention proposal to prevent and reduce cyberbullying during adolescence

The intervention proposal is made up of a program (Cyberprogram 2.0; Garaigordobil and Martínez-Valderrey, 2014a) and a videogame (Cooperative Cybereduca 2.0; Garaigordobil and Martínez-Valderrey, 2016b), which are designed to prevent and reduce cyberbullying during adolescence.

### Objectives of the intervention proposal

The set of activities that make up the intervention to prevent and reduce cyberbullying revolves around 4 large general objectives that include several specific objectives:

1. *To know what bullying and cyberbullying are, to reflect on the people involved in these situations*: (1) Provide specific insight into the concepts of bullying and cyberbullying; (2) Define the three roles involved in the phenomenon (victim, aggressor, observer) and be aware of the behaviors associated with these roles; and (3) Identify and analyze cases of cyberbullying that have occurred or are occurring in the school or outside the school.
2. *To become aware of the harm caused by such behaviors and the severe consequences for all involved:* (1) Promote critical capacity in the face of cyberbullying; (2) Analyze and highlight the feelings of the victims, the aggressors, and the observers; and (3) Foster empathy toward the victim, increasing critical capacity and the capacity to identify the rights and obligations of those involved in cyberbullying behaviors.
3. *To learn guidelines to prevent and deal with these situations: know what to do when one suffers this kind of violence or when observing that someone else is suffering it:* (1) Encourage dialogue as a method of conflict resolution; (2) Develop a sense of shared responsibility; (2) Develop constructive guidelines for each of the roles involved (victim, aggressor, observer); and (3) Provide cyberprotection measures, as a first level of safety.
4. *Promote other related objectives:* (1) Increase the capacity of empathy, in order to put oneself in the place of the other and understand the emotional states of others; (2) Improve intra-group communication, promoting active listening and the expression of ideas, thoughts, and feelings; (3) Develop social skills; (4) Promote an increase in strategies to control anger and impulsiveness in favor of conflict resolution; (5) Enhance the capacity of cooperation among the members of the group; and (6) Encourage the expression of emotions through drama, drawing, etc.

The intervention proposal promotes cognitive restructuring of the roles involved in bullying/cyberbullying, while the modification of cognitions promotes behavioral changes. Victims learn to defend themselves and observers learn to intervene in favor of the victims.

### Features, modules, and activities of the proposal

The intervention program has been designed for use with adolescent groups, but it can also be implemented with youth of older age groups. This experience can be carried out by the teacher-tutor of the group or by the school psychologist.

The program's 25 activities are distributed in 3 intervention modules or axes about bullying and cyberbullying: (1) *Conceptualization and identification of roles* (the activities focus mainly on knowing the characteristics and behaviors of the two phenomena, identifying the various roles involved in these situations); (2) *Consequences, rights, and responsibilities* (the activities promote awareness of the severe consequences of bullying and cyberbullying for victims and aggressors, and the observers' responsibility for the continuation of the situation); (3) *Coping strategies* (the activities teach guidelines to prevent this type of violence and provide strategies to cope with it, both in the role of victim and of observer). The program concludes with the videogame Cooperative Cybereduca 2.0 (see Table 1).

**Table 1:** Modules and activities of the intervention proposal.

**Modules**	**Activities**
Module 1. Conceptualization and identification of roles	The cyberbullying corner Guess the Word 2.0. Collage Who's who? Colored post-its
Module 2. Consequences, rights and responsibilities	Secrets from cyber-rooftops Sexting and false promises Posters Social networks Don't trust completely
Module 3. Coping strategies	Jokes aside Megan Meier and Ryan Halligan Let's talk about Patty Problem-solving: What can victims do? Break the law of silence Responding to aggressors Signing a contract Block Internet bullying Inspector Gadget I see, I see; what do you see? The impact of cyberbullying Photo comic Creating a blog Film-forum Visit to the Museum
Closing of the intervention	Video Game Cooperative Cybereduca 2.0

The activities of the program provide the students with strategies to prevent and deal with bullying in all its forms. The focus of the intervention is: (1) to realize the serious harm produced in the victims in a bullying situation, the adverse effects that it can have in their lives; (2) to be aware of the negative implications, also for the aggressors and observers; and (3) to become aware of the important role that observers play in the maintenance of this situation, as their passivity allows these situations to be perpetuated over time. The intervention sessions mobilize the development of students' social and emotional variables (communication, cooperation, empathy, conflict resolution…), which has the effect of inhibiting violent behavior.

The Cyberprogram 2.0 activities were created *ad hoc*, and the technical specifications of the activities of the program include six informational fields: objectives, description of activity, discussion or debate, materials, time, and group structure. The manual of the program is made up of the technical cards of the activities and the methodology to implement it and assess its impact. The Cyberprogram 2.0 manual also includes a CD that contains all the necessary materials for the development of activities that can be provided by this software, for example, cards to analyze the activities, complementary materials…. As an example, two activities of Cyberprogram 2.0 (Garaigordobil and Martínez-Valderrey, 2014a) are presented. The first one aims to enhance awareness of the victim's suffering, foster empathy with the victim, and identify coping strategies. The second activity aims to identify actions to be carried out by the school to prevent, reduce, and cope with bullying/cyberbullying.

The activity “*Let's talk about Patty*” aims to: (1) analyze the feelings of the victims, the aggressors, and the observers, enhancing empathy for the victims; (2) reflect on the consequences of bullying/cyberbullying for everyone involved; (3) identify positive strategies to cope with bullying situations; and (4) identify bullying situations within the group (face-to-face and/or electronic). The children watch a video in which Lindsay, a girl, reads a composition about another classmate in the assembly hall. http://www.youtube.com/watch?v=bdQBurXQOeQ. The composition refers to the classmate's defects in an insulting and humiliating way. Moreover, the narration is read out loud and in front of a large audience of classmates and teachers who listen in silence. As Lindsay reads the narration, we see Patty's face, distorted by shock, as she hears, one by one, all kinds of critical statements about her. After viewing the video, a large group debate is carried out. The adult will launch questions, for example: What roles are identified in the story? How does the victim feel? How does the aggressor feel? How do the observers feel? and so on. The participants will respond in the whole group to the issues proposed by means of the brainstorming technique, and the adult will summarize and write the main contributions on the blackboard. Secondly, the group is divided into three teams, and each one will receive a card with the word “happy,” “tragic,” or “neutral.” Each team should write down and represent the end of Patty's story, depending on the word they were assigned. The drama will show the situation and the assigned end. At the end of the dramatizations, there is a debate about the different outcomes. The reflection focuses on analyzing the consequences for all those involved: What consequences derive from this type of behavior for all those involved? Why do we say things in virtual environments that we would not dare to say face-to-face? Do we know of any similar case? How can we avoid having offensive attitudes toward others? What should we do in a situation like this, if we are suffering these behaviors by others?.

The activity “*Secrets from cyber-rooftops”* has the following objectives: (1) to identify behaviors that are part of the cyberbullying phenomenon. (2) to raise awareness of the serious consequences of cyberbullying for everyone involved; (3) to reflect on the value of trust in cyber-relations (intimacy and privacy); (4) to analyze the risks of sharing information in cyberspace; (5) to develop a critical capacity for the healthy use of ICT; (6) to promote empathy for cybervictims; and (7) to promote communication, cooperation, and emotional expression. In this activity, firstly, the group members watch a video (http://www.youtube.com/watch?v=97ZBIhvCCEg) in which a teenager spreads intimate information about two classmates, referring to their sexual orientation. The video deals with a situation in which there is a theft of images. The affective, loving relationship between two girls is photographed and broadcast in the whole institute. Subsequently, the students analyze the victims' feelings and the consequences of that behavior for all those involved (because disclosing/revealing/disseminating secrets is an offense that is typified in the penal code). Later, teams of 4–5 students are formed and each team should consider strategies for the victim and/or the observers to cope with the situation, analyze them, and select the strategy they consider the most positive and effective for the victim and/or the observers to deal with the situation. By turns, each team represents the solution, showing the coping strategy they considered the most effective. After finishing the representations, a debate takes place in which the teams report other strategies that were considered positive for victims and observers to deal with that situation.

### Implementation procedure of the intervention proposal

To implement this proposal with a group, a weekly intervention session of approximately 1 h was carried out during a school year. The session is directed by the school psychologist and/or the group's tutor. It takes place in a large, diaphanous classroom, without any obstacles (tables, chairs), which provides a computer with connection to internet, a blackboard, and mats or cushions to sit comfortably on the floor. The group goes to the classroom where the intervention takes place, the participants sit on the floor in a circle, and the session begins. Firstly, the adult briefly explains the objectives of the activity, and usually after watching a short video, instructs the group on how to carry out the activity. Secondly, the group members, distributed in teams, carry out the action cooperatively. Finally, a phase of debate or discussion is conducted about the activity carried out, and the teams' reflections and/or conclusions are shared, scenes are dramatized, and/or the students discuss the products of their activity (e.g., behaviors that they think serve to prevent cyberbullying…). In general, all the intervention sessions follow this structure.

The main techniques of group dynamics used in this proposal include: (1) brainstorming, which is used in many activities that require constructive responses to problems; (2) drama or role-playing of situations in which, for example, constructive ways of coping with bullying/cyberbullying by victims and observers are presented; and (3) the study of real cases of students who suffered bullying/cyberbullying, and this experience caused severe psychological harm and, in some cases, even led them to commit suicide. In the last session, the videogame “*Cooperative Cybereduca 2.0”* is played.

### Cooperative cybereduca 2.0. a videogame to prevent and reduce bullying and cyberbullying

To complement and reinforce the effects of Cyberprogram 2.0, a videogame has been built, “*Cooperative Cybereduca 2.0. A videogame to prevent and reduce bullying and cyberbullying”* (Garaigordobil and Martínez-Valderrey, 2016b), which can be accessed at the following website: http://www.cybereduca.com

The videogame is played online and is free of charge. It is intended to play on computers, with an adult who guides the development of the game and who fosters reflection, although it can also be played independently by the adolescents to whom it is addressed as well as individually. It is multilingual, it can be played in Spanish, Basque, and English and it can be implemented in a variety of contexts: in schools playing with the entire classroom under the teacher's guidance, in groups of leisure time, in the family context, with parents and children playing… The videogame is frequently played in the classroom, as the last activity of Cyberprogram 2.0, as its conclusion and to recall and review all the points reflected on and experienced in the course of its implementation.

This videogame is a Trivial Pursuit consisting of questions and answers that revolve around the topic of bullying and cyberbullying, face-to-face bullying and bullying through ICT (Internet, mobile phone…). This cybernetic trivial pursuit is organized around a history of fantasy, a comic that guides the game and has three features: it is a cooperative, constructive, and non-sexist game. Cybereduca 2.0 is a *cooperative game*. Cooperative games promote communication, increase prosocial behavior, enhance group cohesion, and improve one's self-concept and of that of others. Cybereduca 2.0 is literally a *constructive game*. The characters are construction guilds whose goal is to use their cooperative skills to rebuild fantasy worlds. The guilds of Cybereduca 2.0 do not wield weapons, but instead tools, and they do not fight any battles, but instead, they cooperate with each other to achieve their common goals and thus restore peace and coexistence. In addition, the correct answers to the questions of each team are integrated into a global score for all the players. Cybereduca 2.0 is a *non*-*sexist game* that presents the same number of male and female characters, equitably distributed throughout the game. The collective guilds are represented by a girl-and-boy team. Both team members have similar physical and psychological profiles, not differential according to sex, and they use the same kind of clothing without any variation. The unitary guilds are represented either by an androgynous character or by a non-stereotyped feminine one.

The game starts with an earthquake in the city of Zanthia, where the characters are found. The earthquake causes the opening of a vortex, and all the characters/guilds fall into the vortex into cyberspace. The characters/guilds represent the five roles involved in a bullying/cyberbullying situation: (1) Aggressors (skull stonemasons' guild: tough, insensitive guys, who play practical jokes on others…); (2) Victims (solitary painters' guild: sensitive, the target of the skull stonemason's jokes, solitary…); (3) Defending observers of the victims (justice engineers' guild: they tend to get along with everyone, but they do not tolerate injustice, if someone is behaving badly, they tell them to their face…); (4) Observers who support the aggressors (giggling carpenters' guild: sociable and fun, they like to be part of the group, they do not tend to play jokes on the victims, but if others do so, they laugh); and (5) Passive observers who do not intervene (impassive plumbers' guild: they go their own way, they don't argue with anyone, but remain aloof from problems).

The guilds (players/teams) must perform challenging actions (open doors, freeing missing characters) and rebuild worlds (Loot Bay, Gadget Villa, Dragon Nest, Flying High, and City of Zanthia). For this purpose, they must cooperate by answering questions because, in essence, Cybereduca is a game of trivial pursuit. The complete set contains 120 questions that revolve around 5 topics: cyberphenomena, computer technology and security, cybersexuality, consequences of bullying/cyberbullying, and coping with bullying/cyberbullying.

*Topic 1. Cyberphenomena:* In this topic, the contents of the questions help to identify and define bullying, cyberbullying, and other cyberphenomena related to the use of the mobile, internet, ICT, such as grooming, nomophobia, cyberbaiting, flaming, griefing, trolling, outing, cyberstalking, phishing… For example: The new phenomenon consisting of a compulsion that affects mobile Internet users, and characterized by having an irrational fear of going out without one's smartphone, is called: (A) Phobiaphone. (B) *Nomophobia*. (C) Monophobia.
*Topic 2. Computer technology and Security:* The questions in this topic, on the one hand, help to clarify computer concepts (firewall, cookie, blogger, antivirus, router, spam, anti-pop-up programs …), provide data on protection rules and the safe use of ICT (mobile, internet…), and, on the other hand, they identify risky behaviors and teach ethical standards of behavior in social networks and cyberspace. For example: If we suspect that someone is accessing our e-mail account or impersonating our identity, we should: (A) Delete the account and create a new one. (B) Change the password. (C) *Report it to the police*.
*Topic 3. Cybersexuality*: The questions associated with this topic, on the one hand, help to identify, prevent, and deal with sexting and, on the other hand, they foster reflecting on various sexual behaviors that are performed using ICT and that have very negative consequences, such as sextortion, grooming, sexual abuse, etc. For example: Sending sexually explicit images among people of the same age isn't a problem: (A) No, because they are people of the same age; (B) It depends on whether there's an affective relationship between those people; (C) *It's a problem indeed, because the image becomes undeletable and can be disclosed to other people*.
*Topic 4. Consequences of bullying/cyberbullying*: In this topic, the contents of the questions allow the identification of the consequences of face-to-face and electronic bullying for the victims, the aggressors, and the observers (emotional, social, and intellectual effects…), enhancing the development of empathy for the victims. For example: What is one of the effects of cyberbullying on observers, as bystanders in cyberbullying situations? (A) None, because they have nothing to do with it. (B) None, provided that they don't get involved. (C) *They may become unsupportive people, insensitive toward other people's feelings*.
*Topic 5. Coping with bullying/cyberbullying:* In this topic, problematic situations are presented, showing appropriate behaviors to deal with situations of bullying, cyberbullying and other events associated with the use of ICT, from the perspective of the victims, the observers, and the aggressors. For example: Laura and Marta were best friends “forever” and they shared everything, even their e-mail and social network passwords. One day, they quarreled and stopped being friends. Marta used Laura's password to access her social network account, and she sent offensive messages to all of her contacts, pretending to be Laura. What advice would you give to Laura? (A) Don't tell anybody anything and send an apology to all your contacts. (B) Remove all your contacts. (C) *Report the issue on your social network, make Marta realize the consequences of her behavior, and change your password regularly*.

In addition to the cards of these five areas, in which there is always a correct answer, the game includes some complementary cards that require performing representations and cooperating. Dramatic actions encourage the players to represent positive and negative emotions (sadness, happiness, anger, fear…), and to perform prosocial actions in which they must cooperate with other team members (for example, giving a hug, cheering, singing a song…).

In the dynamics of this game, the teams answer questions that allow them to open doors, free the videogame characters, and rebuild worlds to return to the city of Zanthia, always with the cooperation of all the players. The game is won when they all achieve the goals after answering questions and rebuilding the worlds through cooperative construction actions. This allows them to return to the city of Zanthia in which from that moment, a positive social climate of harmony and camaraderie among the guilds will reign.

## Empirical assessment study of the intervention proposal: methodology and results

This intervention proposal is based on evidence, that is, it has been validated experimentally. The evaluation of the effects of Cyberprogram 2.0 was conducted by applying Cyberprogram 2.0 and the game Cybereduca 2.0 in a similar, non-digital format because the Cybereduca game was transformed into a videogame later on (2016). The results of the intervention have been recently published, so below, we present a synthesis of the empirical study carried out regarding its methodology and the results obtained.

### Participants

This study was carried out with a sample of 176 adolescents, aged between 13 and 15 years, who studied Compulsory Secondary Education (grades 9 and 10). Out of the total sample, 93 (52.8%) were assigned to the experimental condition and 83 (47.2%) to the control condition. Of the sample, 25% were 13 years old, 48.9% were 14, and 26.1% were 15. The study was carried out in three schools of Gipuzkoa (northern Spain) of diverse socioeconomic-cultural level. Of the students, 44.3% attended public schools and 55.7% a private center. A random sampling technique was used to select the sample, taking into account the list of schools in Gipuzkoa and the type of center (public-private).

### Procedure

The study used a quasi-experimental, repeated pretest-posttest measures design with a control group. To carry out the research, first, the schools were randomly selected, and we described the project to the principals and requested their collaboration. We provided informed consent forms for the parents and participants of the schools whose principals agreed to participate. After the consent forms had been signed, the research team applied the pretest assessment in the schools (see Table 2). In each school, some classrooms were randomly assigned to the experimental condition and others to the control group. The intervention was implemented with the experimental group during the school year, while the control students carried out the usual activities of the school tutoring program. Subsequently, the posttest assessment was done, using the same tools as at pretest. The study respected the ethical values required in research with humans, and received the favorable report of the Ethics Committee of the University of the Basque Country (CEISH/112/2012).

**Table 2:** Pretest-posttest evaluation instruments.

**Instruments**	**Variables**	**Task**	**Psychometric data: reliability and validity**
Cyberbullying: Screening of Peer Harassment (Garaigordobil, 2013)	*Bullying and Cyberbullying:* Victimization Perpetration Observation Cybervictimization Cyberperpetration Cyberobservation	Report if they have suffered, carried out, and seen bullying behaviors (physical, verbal, social, and psychological aggressive behaviors) and 15 cyberbullying behaviors in the past year on a Likert scale ranging from 0 to 3	Bullying: Reliability: Total (α = 0.81), Victimization (α = 0.70), Perpetration (α = 0.71), Observation (α = 0.80). Cyberbullying: Reliability: Total (α = 0.91), Cybervictimization (α = 0.82), Cyberperpetration (α = 0.91) Cyberobservation (α = 0.87). Factor analysis confirmed a 3-factor structure (victims, aggressors, observers in the Bullying and Cyberbullying Scales, which explain, respectively, 57.89 and 40.15% of the variance).
CUVE- R. Revised Questionnaire of School Violence; (Álvarez-García et al., 2011)	Diverse types of school violence: teachers' violence toward students, students' physical and verbal violence, social exclusion, disruption in the classroom, violence by means of ICT	31 statements that refer to face-to-face and ICT bullying behaviors, and they must indicate the frequency with which they observed them happening, rating this frequency on a scale from 1 to 5	Reliability: α = 0.92. Validity: Confirmatory factor analysis evidences the six factors.
AVE. Bullying and School Violence Questionnaire (Piñuel and Oñate, 2006)	*Global bullying index*	50 statements on behaviors of harassment, intimidation, and threats to integrity, coercion, social exclusion…. The teenager reports the frequency with which what is described in the sentence has happened to him/her.	Reliability: α = 0.95.
CAPI-A. Adolescents' Premeditated and Impulsive Aggressiveness Questionnaire (Andreu, 2010)	*Aggressiveness:* Impulsive Premeditated	24 statements about ways of thinking, feeling, or acting that participants self-apply and rate the degree of agreement with the contents on a 1–5 scale	Reliability: Premeditated Aggressiveness α = 0.83; Impulsive Aggressiveness α = 0.82. Convergent validity: significant correlations between impulsiveness and reactive aggressiveness; premeditated aggression and proactive aggressiveness.
AECS. Attitudes and Social Cognitive Strategies Questionnaire (Moraleda et al., 2004)	*Social Behaviors:* Social conformity Help-collaboration Self-assurance-firmness Prosocial leadership Aggressiveness-stubbornness Dominance Apathy-withdrawal Social anxiety	71 statements about positive and negative social behaviors that participants self-apply and rate the extent to which they carry out the described acts on a 1–7 scale.	Reliability: Positive Behaviors α = 0.75; Negative Behaviors α = 0.85. Criterial validity: social adaptation. Convergent validity: correlation analysis between the Criteria Socialization scores of the BAS.
RSE. Rosenberg Self-Esteem Scale (Rosenberg, 1965)	*Self-Esteem*	10 statements about self-esteem that participants self-apply and rate their degree of agreement on scale of 1–4.	Reliability: α = 0.74. Validity: unidimensional measure of self-esteem found in numerous studies.
CONFLICTALK. Conflictalk. An instrument for measuring youth and adolescent conflict-management message styles (Kymsey and Fuller, 2003)	*Conflict-management message styles*: Aggressive Cooperative Avoidant	18 statements about ways to resolve conflicts. They should rate each statement on a scale of 1 to 5 (“I never say things like that”/"I almost always say things like that”)	Reliability: Cooperative α = 0.87; Aggressive α = 0.81; Avoidant α = 0.63. Validity: positive correlations between communication skills and cooperative resolution; and negative correlations with aggressive and avoidant resolution.
IECA Index of Empathy for Children and Adolescents (Bryant, 1982)	Empathy	22 statements about empathic behaviors and feelings that participants self-apply and rate the degree of agreement on a scale of 1–7.	Reliability: α = 0.68 and α = 0.79. Validity: positive correlations with empathy of other scales and negative with antisocial behavior

### Instruments

In order to measure the dependent variables, before and after implementing the intervention, the experimental and control students both filled in eight assessment instruments (see Table 2).

### Data analysis

After verifying the basic assumptions, to assess the program's effect on the dependent variables, firstly, we carried out descriptive analyses (means and standard deviations), and univariate and multivariate analyses of variance (ANOVA, MANOVA) with the pretest scores obtained on the eight assessment instruments, by the experimental and control participants. Secondly, we carried out descriptive analyses and analyses of covariance of the pretest-posttest differences (ANCOVA, MANCOVA) using the pretest differences between the two conditions as covariate, thereby determining the intervention's impact. In addition, we calculated the effect size (Cohen's *d*: small < 0.50, moderate 0.50–0.79, large ≥0.80) of each variable, at pretest, and in the pretest-posttest differences. Complementary, we calculate the effect size (Eta square) for the groups of variables (Cohen's values: from 0.01 to 0.04 are judged to be small, 0.04 to 0.14 moderate, and greater than 0.14 large). The statistical analyses were performed with the SPSS 21.0 program.

## Results

The results obtained have shown that the proposal significantly promoted the following changes (see Tables 3, 4) (Garaigordobil and Martínez-Valderrey, 2014b, 2015a,b, 2016a):

1. *A reduction of bullying and cyberbullying behaviors*: These behaviors were significantly reduced in the experimental group compared to the control group, and this decrease was confirmed in all three roles, according to the information provided by victims, aggressors, and observers. The analysis of the change in the two conditions showed that those who participated in the intervention decreased the indicators of victimization, aggression, and aggressive-victimization both in presential or face-to-face bullying and in cyberbullying.
2. *Improved perception of school violence:* Compared with the control group, the experimental participants improved their perception of school violence, both of peer violence through diverse aggressive physical, verbal, social, and technological behaviors, as well as of teachers' violence toward students.
3. *A decrease in aggressiveness:* Aggressive behaviors, both those that express impulsiveness and anticipatory or premeditated aggressiveness, decreased in the experimental group significantly more than in the control group. The evaluation confirmed that those who participated in the intervention reduced their impulsive aggressive behaviors (with negative emotional arousal, in response to a perceived provocation, reactive, and hostile), and their premeditated aggressive behaviors (behaviors with a goal, unprovoked, with no negative emotional arousal, seeking the consequences of violence, pro-active, and instrumental).
4. *An increase of all the positive social behaviors assessed*: *social conformity* (compliance with social rules and norms that facilitate coexistence and mutual respect); *help and collaboration* (giving, sharing, cooperating, reinforcing and stimulating the work of others, reaching solutions by consensus); *self-confidence and firmness* (trust in their abilities to achieve the objectives pursued, assertiveness in defense of their rights, coping with problems); and *prosocial leadership behaviors* (tendency to take the initiative, to plan prosocial activities, with a spirit of service). These behaviors increased significantly more in the experimental group compared with the control group.
5. *An improvement of the capacity to resolve interpersonal conflicts*: The experimental group showed a reduction in the use of negative conflict resolution strategies (aggressive, avoidant) and an increase in the positive-cooperative ones. Those who had participated in the intervention learned to use more constructive strategies to solve interpersonal conflicts.
6. *An increase in self-esteem*: Global feelings of self-appraisal increased significantly more in the experimental group compared with the control group.
7. *An improvement of the capacity for empathy:* The ability to understand the emotional states of other human beings increased significantly more in those who participated in the antibullying intervention.

**Table 3:** Means, Standard Deviations, Results of the Pretest ANOVAs, of Pretest-Posttest ANCOVAs, and Effect Size (d) in all variables in Experimental and Control groups.

	**Pretest**				**Pretest-Posttest Differences**				**Pretest ANOVA**			**Pretest-Posttest ANCOVA**		
	**Experimental**		**Control**		**Experimental**		**Control**							
	***M***	***SD***	***M***	***SD***	***M***	***SD***	***M***	***SD***	***F*_(1, 174)_**	***p***	***d***	***F*_(1, 174)_**	***p***	***d***
Victimization of bullying	0.75	1.10	0.55	1.01	−0.18	1.12	0.39	1.90	1.53	0.217	0.18	5.22	0.024	0.36
Perpetration of bullying	1.57	1.88	0.54	0.86	−0.87	1.91	0.39	1.41	20.80	0.000	0.70	5.46	0.021	0.75
Observation of bullying	3.73	2.69	2.16	2.18	−1.11	2.93	0.27	2.63	17.86	0.000	0.64	1.25	0.264	0.49
Aggressive-victimization (bullying)	2.32	2.36	1.10	1.51	−1.06	2.38	0.77	3.04	16.40	0.000	0.61	6.87	0.010	0.67
Victimization of cyberbullying	1.20	3.26	0.90	3.41	−1.10	3.27	0.61	4.64	0.35	0.551	0.10	13.89	0.000	0.42
Perpetration of cyberbullying	0.69	1.43	0.25	0.93	−0.65	1.41	0.45	1.73	5.52	0.020	0.36	14.55	0.000	0.69
Observation of cyberbullying	3.29	3.24	2.60	2.94	−0.38	3.66	0.77	4.97	2.14	0.145	0.22	3.63	0.058	0.27
Aggressive-victimiz. (cyberbullying)	1.89	3.80	1.16	3.69	−1.75	3.78	1.04	6.01	1.68	0.196	0.19	14.89	0.000	0.56
Bullying victimization	4.84	6.98	3.67	5.15	−3.16	6.52	2.41	10.15	1.55	0.215	0.19	19.50	0.000	0.65
Teachers' Violence toward Students	16.56	6.48	12.67	4.79	−1.96	5.70	1.91	4.79	20.00	0.000	0.68	7.43	0.007	0.73
Students' Physical Violence	13.22	4.56	10.55	3.77	−1.59	4.33	1.12	4.08	17.48	0.000	0.63	4.34	0.039	0.64
Students' Verbal Violence	15.16	4.58	11.66	3.56	−2.75	4.71	1.46	3.93	31.43	0.000	0.85	11.49	0.001	0.97
Social Exclusion	5.40	1.88	4.81	1.79	−0.98	1.86	0.49	1.90	4.50	0.035	0.32	21.55	0.000	0.78
Disruption in the Classroom	8.26	2.94	7.11	2.71	−0.35	3.14	0.54	2.77	7.18	0.008	0.40	0.09	0.763	0.30
ICT Violence	9.63	4.08	7.45	2.18	−2.41	3.04	1.00	3.46	18.97	0.000	0.66	33.56	0.000	1.04
Premeditated Aggressiveness	29.05	7.56	25.45	6.43	−6.09	10.38	1.38	8.66	11.34	0.001	0.51	31.14	0.000	0.78
Impulsive Aggressiveness	32.38	10.25	30.18	8.64	−9.21	10.46	−1.76	8.02	2.30	0.131	0.22	34.85	0.000	0.79
Social conformity	39.56	11.48	40.24	10.74	6.82	13.55	−0.81	13.47	0.16	0.686	0.06	17.16	0.000	0.56
Help-collaboration	49.99	12.46	50.50	11.02	2.86	12.27	−1.96	12.37	0.08	0.777	0.04	8.38	0.004	0.39
Self-assurance-firmness	52.98	12.18	52.76	10.42	2.84	13.22	−3.91	14.89	0.01	0.899	0.01	13.46	0.000	0.47
Prosocial leadership	17.30	5.91	17.60	5.62	0.56	5.44	−1.39	6.55	0.11	0.736	0.05	4.06	0.045	0.32
Aggressiveness-stubbornness	25.12	8.72	24.18	11.27	−2.65	9.20	−2.05	13.12	0.37	0.540	0.09	0.00	0.980	0.05
Dominance	12.77	8.48	13.17	8.28	−1.35	7.72	−1.52	8.91	0.10	0.753	0.04	0.00	0.931	0.02
Apathy-withdrawal	23.10	9.73	23.06	8.78	−2.96	8.75	−0.91	10.13	0.00	0.978	0.00	3.12	0.079	0.21
Social anxiety	16.53	8.82	19.17	9.12	−0.65	8.93	−0.76	9.04	3.71	0.056	0.29	1.47	0.227	0.01
Avoidant Conflict Resolution	12.02	4.79	12.12	4.25	−1.25	5.73	0.35	4.66	0.02	0.885	02	7.32	0.007	0.30
Aggressive Conflict Resolution	10.59	4.65	9.45	3.28	−2.51	4.22	−0.02	3.69	3.41	0.066	0.28	16.89	0.000	0.65
Cooperative Conflict Resolution	18.26	8.11	17.71	6.51	2.35	8.09	−0.04	6.55	0.24	0.622	0.07	10.74	0.001	0.33
Self-esteem	30.80	6.79	31.67	5.92	4.02	7.41	−0.81	5.97	0.79	0.374	0.13	30.07	0.000	0.71
Capacity for empathy	96.15	21.22	97.48	18.42	7.20	17.43	0.60	11.69	0.19	0.664	0.06	11.77	0.001	0.28

*d, Cohen's effect size. Experimental n = 93, Control n = 83*.

**Table 4:** Results of the Pretest MANOVAs, of Pretest-Posttest MANCOVAs, and Effect Size (η^2^) in all grouped variables, between experimental and control groups.

	**Pretest MANOVA & Effect Size**					**Pretest-Posttest MANCOVA & Effect Size**				
	**Λ**	***F***	***p***	**η^2^**	***r***	**Λ**	***F***	***p***	**η^2^**	***r***
SCREENING. Bullying & Cyberbullying	0.852	4.87	<0.001	0.148	0.38	0.834	5.58	<0.001	0.087	0.29
CUVE-R. School Violence	0.798	7.12	<0.001	0.202	0.44	0.664	12.09	<0.001	0.336	0.57
CAPI-A. Aggressiveness	0.936	5.90	<0.01	0.064	0.25	0.859	13.99	<0.001	0.141	0.37
AECS. Social Behavior	0.957	0.91	>0.05	0.043	0.20	0.847	3.45	<0.05	0.153	0.39
CONFLICTALK. Conflict Resolution	0.972	1.62	>0.05	0.028	0.16	0.849	9.87	<0.001	0.151	0.38

*Λ, Wilks' Lambda; η^2^, Eta squared effect size*.

## Discussion

The results provide evidence of the effectiveness of the program, validate the intervention proposal, and, as a whole, allow us to emphasize the importance of implementing programs to prevent cyberbullying and other negative cyberphenomena (nomophobia, sexting, sextortion, grooming…). The study provides a tool of effective psycho-educational intervention to prevent and reduce cyberbullying during adolescence. The use of self-reports, with the inherent bias of social desirability, is a limitation of the study.

These results are coherent with other studies showing the efficacy of antibullying interventions to decrease aggressiveness (McMahon et al., 2000; Fonagy et al., 2005; Twemlow et al., 2005) and violent peer behaviors of victimization and perpetration, either face-to-face (Olweus, 2004; Fekkes et al., 2006; Gollwitzer et al., 2006; Milton and O'Moore, 2008; Kärnä et al., 2011; Palladino et al., 2012; Williford et al., 2012) or electronic (Doane, 2011; Lee et al., 2013; Williford et al., 2013; Chaux et al., 2016). In addition, the results point in the same direction as other studies showing the efficacy of antibullying programs to increase prosocial behaviors (Grossman et al., 1997; Gini, 2004), social competence (Leadbetter et al., 2003), and self-concept—self-esteem (DeRosier and Marcus, 2005; Rawana et al., 2011).

Among the factors that can explain the positive results of the intervention are the characteristics of the activities, as they promote: (1) empathy toward the victim (understanding the victim's feelings and the severe harm involved in bullying/cyberbullying for its victims); (2) analysis of the consequences for all those involved in bullying/cyberbullying; and (3) mobilization of the observers to defend the victim and denounce what they are observing. The activities included in this proposal stimulate affective-emotional and social development processes (e.g., the ability to dialogue rationally, two-way communication, assertiveness, prosocial behavior, empathy, constructive conflict resolution…), which play an important role in reducing violent behavior. These activities create a positive climate in the group, which promotes coexistence, increases prosociality, and reduces violence.

## Author contributions

MG: has designed the research and she has supervised the application of the instruments and the implementation of the program. She has carried out the data analysis and has written the article. VM-V: has applied the evaluation instruments and has implemented the program. She has collaborated in the design of the program and in the analysis of data.

### Conflict of interest statement

The authors declare that the research was conducted in the absence of any commercial or financial relationships that could be construed as a potential conflict of interest.
